# Unravelling the Difference Between Men and Women in Post-CABG Survival

**DOI:** 10.3389/fcvm.2022.768972

**Published:** 2022-04-13

**Authors:** Amand F. Schmidt, Saskia Haitjema, Ulrik Sartipy, Martin J. Holzmann, David J. Malenka, Cathy S. Ross, Wiek van Gilst, Jean L. Rouleau, Annelijn M. Meeder, Robert A. Baker, Hiroki Shiomi, Takeshi Kimura, Lavinia Tran, Julian A. Smith, Christopher M. Reid, Folkert W. Asselbergs, Hester M. den Ruijter

**Affiliations:** ^1^Department of Cardiology, Division Heart and Lungs, University Medical Center Utrecht, Utrecht, Netherlands; ^2^Institute of Cardiovascular Science, Faculty of Population Health, University College London, London, United Kingdom; ^3^Department of Clinical Chemistry and Haematology, University Medical Center Utrecht, Utrecht, Netherlands; ^4^Department of Molecular Medicine and Surgery, Karolinska Institutet, Stockholm, Sweden; ^5^Department of Cardiothoracic Surgery, Karolinska University Hospital, Stockholm, Sweden; ^6^Department of Medicine, Functional Area of Emergency Medicine, Karolinska University Hospital, Karolinska Institutet, Stockholm, Sweden; ^7^The Geisel School of Medicine at Dartmouth, The Dartmouth Institute for Health Policy and Clinical Practice, Lebanon, NH, United States; ^8^Department of Medicine, Heart and Vascular Center, Dartmouth-Hitchcock Medical Center, Lebanon, NH, United States; ^9^Department of Cardiology, University Medical Center Groningen, University of Groningen, Groningen, Netherlands; ^10^Montreal Heart Institute, University of Montreal, Montreal, QC, Canada; ^11^Department of Anesthesiology, University Medical Center Utrecht, Utrecht, Netherlands; ^12^Quality and Outcomes, Cardiothoracic Surgical Unit, Flinders Medical Centre, Adelaide, SA, Australia; ^13^Perfusion Service, Cardiothoracic Surgical Unit, Flinders Medical Centre, Adelaide, SA, Australia; ^14^Department of Surgery, College of Medicine and Public Health, Flinders University, Adelaide, SA, Australia; ^15^Department of Cardiovascular Medicine, Kyoto University Graduate School of Medicine, Kyoto, Japan; ^16^School of Public Health and Preventive Medicine, Monash University, Melbourne, VIC, Australia; ^17^Department of Surgery, School of Clinical Sciences at Monash Health, Monash University, Clayton, VIC, Australia; ^18^Department of Cardiothoracic Surgery, Monash Health, Clayton, VIC, Australia; ^19^School of Public Health, Curtin University, Perth, WA, Australia; ^20^Health Data Research UK, Institute of Health Informatics, University College London, London, United Kingdom; ^21^Laboratory of Experimental Cardiology, University Medical Center Utrecht, Utrecht, Netherlands

**Keywords:** sex, gender, CABG, outcome, prognosis, atherosclerosis

## Abstract

**Objectives:**

Women have a worse prognosis after coronary artery bypass grafting (CABG) surgery compared to men. We sought to quantify to what extent this difference in post-CABG survival could be attributed to sex itself, or whether this was mediated by difference between men and women at the time of intervention. Additionally, we explored to what extent these effects were homogenous across patient subgroups.

**Methods:**

Time to all-cause mortality was available for 102,263 CABG patients, including 20,988 (21%) women, sourced through an individual participant data meta-analysis of five cohort studies. Difference between men and women in survival duration was assessed using Kaplan–Meier estimates, and Cox’s proportional hazards model.

**Results:**

During a median follow-up of 5 years, 13,598 (13%) patients died, with women more likely to die than men: female HR 1.20 (95%CI 1.16; 1.25). We found that differences in patient characteristics at the time of CABG procedure mediated this sex effect, and accounting for these resulted in a neutral female HR 0.98 (95%CI 0.94; 1.02). Next we performed *a priori* defined subgroup analyses of the five most prominent mediators: age, creatinine, peripheral vascular disease, type 2 diabetes, and heart failure. We found that women without peripheral vascular disease (PVD) or women aged 70+, survived longer than men (interaction *p*-values 0.04 and 6 × 10^–5^, respectively), with an effect reversal in younger women.

**Conclusion:**

Sex differences in post-CABG survival were readily explained by difference in patient characteristics and comorbidities. Pre-planned analyses revealed patient subgroups (aged 70+, or without PVD) of women that survived longer than men, and a subgroup of younger women with comparatively poorer survival.

## Introduction

Sex differences in coronary artery disease (CAD) have been established with women presenting distinctly from men, with more stable and diffuse atherosclerotic disease (1). Plaque erosions, likely driven by sex hormones, are commonly observed in younger women who experienced a fatal myocardial infarction (MI), whereas plaque ruptures have been more frequently described in men. At an older age, differences between men and women in pathophysiology of atherosclerosis are less pronounced, and women present with more complicated risk factor combinations (2, 3). Cardiac surgery is one of the main therapeutic options when cardiac atherosclerotic complications occur. For both short-term and long-term survival, sex differences after coronary artery bypass grafting (CABG) have been reported with women having a poorer prognosis than men (4, 5).

**FIGURE 1:**
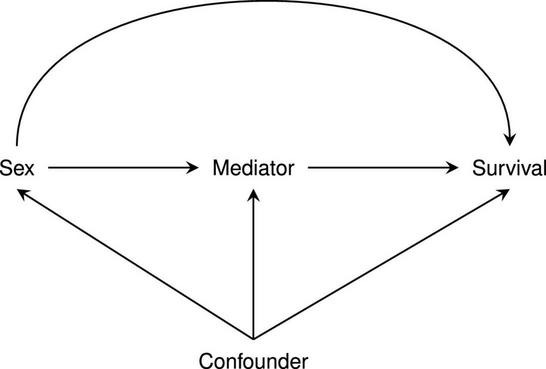
Diagram differentiating confounding from mediation.

While sex often causes confounding, sex itself is determined at conception and therefore sex associations are robust to most forms of confounding (defined as a common cause of both exposure and outcome; Figure 1). Nevertheless, men and women are known to present with different patient characteristics at the time of CABG, and it remains to be seen if post-CABG survival difference can be attributed to sex itself, or rather to risk-factor mediation. Here we differentiate between mediation and confounding, by following the standard definition specifying mediation as any variable positioned between sex and survival in a potential causal chain: sex → mediator → survival. Identification of mediators is especially relevant because, unlike sex, many mediators can be modified or prevented e.g., incidence of diabetes, which can lead to actionable insights to diminishing sex differences in post-CABG survival.

We initiated the “LOng-Term outcome following coronary arTERy bYpass surgery in women” (LOTTERY) collaboration to quantify to what extent patient characteristics at the time of CABG explained the difference in survival between men and women, and additionally explored if such effects were stable across pre-planned subgroup analyses constituting the major mediating factors.

## Materials and Methods

### Data Sources and Collected Patient Information

The LOTTERY collaboration included 56,990 patients from the Australian ANZSCTS database (6), 2,172 Japanese patients from the CREDO-Kyoto (7), 32,276 patients from the Swedish SWEDEHEART registry (8, 9), 8,274 patients from the American NNECDSG (10) (United States), and 2,551 lower-risk patients from the multinational IMAGINE trial (11). This study was approved by local institutional review boards, with participants giving informed consent; the ANZSCTS operates under an opt-out consent model.

The following patient information, at the time of CABG, was available: sex, age (years), body mass index (BMI), left ventricular ejection fraction (%), creatinine level (μmol/L), number of diseased vessels, whether the procedure was performed on or off-pump, number of grafts, previous myocardial infarction (MI), a history of: hypertension, stroke, or atrial fibrillation, and the presence of: type 2 diabetes, chronic obstructive pulmonary disease, heart failure, peripheral vascular disease, or kidney failure. Unit difference between local registrations were resolved, however, due to limitations of the data (e.g., structured data as opposed to unstructured free-text) local registry definitions were used for categorical variables.

### Statistical Analyses

Study and sex stratified cumulative (all-cause) mortality rates were calculated using the Kaplan-Meier estimator, with difference tested using a log-rank test. 95% confidence intervals (95%CI) were estimated for the first mortality-decile (the time 10% of subjects had perished); mortality rates remained below 50% making the more traditional median survival unavailable. Pairwise Spearman’s correlation coefficients were estimated to quantify the interdependence of patient characteristics at the time of CABG.

To further quantify and explore the association of sex with all-cause mortality we estimated hazard ratios (HRs) using semi-parametric, study-stratified, Cox proportional hazard models. The initial (crude) model regressed the time to all-cause mortality on sex, in a subsequent (adjusted) model, sex-related differences in patient characteristics were accounted for. Variables were included if they were recorded in at least 90% of the study-subjects (see Supplementary Appendix Table 1), resulting in study specific models. Here variables were selected simply based on their availability (lack of missing data), and not necessarily because we felt these were the most relevant mediators.

Study-specific adjustment models, conditioning on a different subset of variables, may possibly induce (instead of decrease) between study differences. Furthermore, unless the observed data is missing completely at random (12), excluding missing data may induce (selection) bias. We analytically accounted for these *potential* short-comings by performing multi-level multiple imputation (13), using MICE (multivariate imputation by chained equations) with 15 imputation sets. These imputed data were used in a second adjustment model (subsequently referred to as “imputed” model) including *all* the above described variables; with results combined using Rubin’s rules.

To assess mediation, we used the “imputed” model and performed a leave-one-out analysis where each variable was sequentially removed, and put back into the model. Mediation was quantified as the *percentage* change in the female sex HR, conditional on *all* remaining patient characteristics. In pre-planned analyses we explored whether the sex association on survival was modified by introducing an interaction term in the imputed model between sex and one of the five mediators eliciting the largest percentage change. Subgroup specific effects were illustrated irrespective of interaction significance.

Next, we explored possible violations of the proportional hazard assumption (where the sex HR would vary across time) by correlating the Schoenfeld residuals with time. In a similar vein we performed a “landmark” analysis, focussing on participants with follow-up of a least 30-days.

Throughout, generalizability was assessed using heterogeneity statistics: Q-test and the I-squared statistic with a one-sided 97.5% confidence interval; with random effects estimates are presented in Supplementary Appendix Table 4.

## Results

Of the 102,263 included patients, 20,988 (21%) were women, the median age was 66.00 (IQR 59.00, 73.00) for men and 70.00 (IQR 62.00, 76.00) for women. On average 31.0% (25,169) men had T2DM at the time of CABG, compared to 38.7% (8,127) women, for PVD this was 88.3% (71,739) and 86.3% (18,115), respectively; see additional patient characteristics provided in Supplementary Appendix Table 2 and Supplementary Appendix Figures 1–3.

**FIGURE 2:**
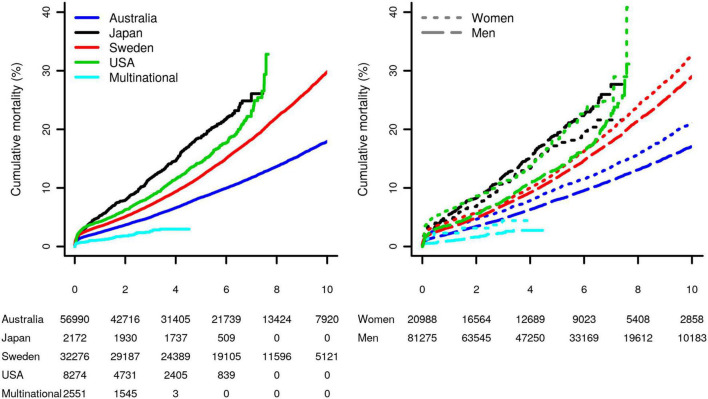
Study-specific Kaplan–Meier curves of the post-CABG cumulative mortality, for the overall sample, as well as stratified by sex. Results were stratified by study as well as by sex (difference between survival curves in the left and right plots were significant, with the both *p*-values <2 × 10^–16^). Subjects at risk are shown in the bottom margin.

During a median follow-up of 5.1 years (interquartile range [IQR]: 2.4, 7.9), 13,598 (13%) patients died: 10,262 (13%) men and 3,336 (16%) women (*p*-value < 2 × 10^–16^). The study specific mortality rates differed considerably between studies (*p*-value < 2 × 10^–16^, left panel Figure 2). The *difference* in sex-specific survival was generally more comparable (Figure 2, right panel), where women had a higher mortality rate after CABG compared to men. After 4.17 years (95%CI 4.17; 4.50) about 10% of the women had died, whereas in men this took 5.04 years (95%CI 4.93; 5.16); see Figure 2 and Supplementary Appendix Table 3.

**FIGURE 3:**
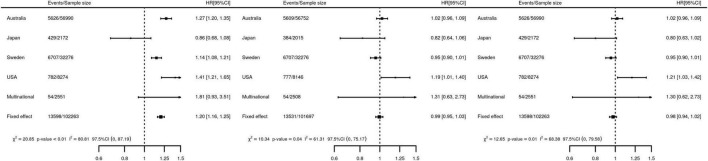
Forest plots of the association of female sex with the time to post-CABG mortality. **Left** panel, a *crude* Cox PH model, regressing the time to event on sex, without accounting for difference in risk factors of post-CABG survival. **Middle** panel, a *study-specific adjusted* model accounting potential risk factors observed for more than 90% of the subjects. Specifically, five covariate models were meta-analyzed where the sex variable was adjusted for the following variables: (I) United States, NNECDSG covariates: off-pump, age, BMI, hypertension, T2DM, creatinine, MI, Kidney disease, number of DV, HF, AF, COPD, PVD, LVEF. (II) Japan, CREDO-Kyoto covariates: off-pump, age, BMI, hypertension, T2DM, creatinine, MI, Kidney disease, number of DV, Stroke, HF, COPD, PVD, LVEF (III) Swedish, SWEDEHEART covariates: off-pump, age, BMI, hypertension, T2DM, creatinine, MI, Stroke, HF, AF, COPD, number of graft PVD. (IV) Australia, ANZSCTS covariates: off-pump, age, BMI, hypertension, T2DM, creatinine, MI, number of DV, Stroke, HF, AF, COPD, number of graft, PVD. (V) Multinational, IMAGINE trial covariates: off-pump, age, BMI, hypertension, T2DM, creatinine, MI, number of DV, Stroke, AF, number of graft, PVD. **Right** panel, multi-level, multiply *imputed* Cox PH model adjusting for age, BMI, left ventricular ejection fraction, creatinine, number of grafts, number of diseased vessels, whether the procedure was performed on or off-pump, previous MI, history of: hypertension, stroke, AF, T2DM, COPD, HF, PVD, and kidney failure. HR, hazard ratio; 95%CI, 95% confidence interval; heterogeneity statistics include Q-tests (χ^2^) and *I*^2^ as well one-sided 97.5% confidence intervals. Random effects estimates are presented in Supplementary Appendix Table 4.

To quantify the difference in post-CABG survival female HR were estimated using a crude model: 1.20 (95%CI 1.16; 1.25, Figure 3). Next, we adjusted for difference between men and women in risk factors for post-CABG survival (adjusted model, middle panel Figure 3), which diminished differences in survival between men and women: HR 0.99 (95%CI 0.95; 1.03). Imputing missing data resulting in a similar HR: 0.98 (95%CI 0.94; 1.02).

**FIGURE 4:**
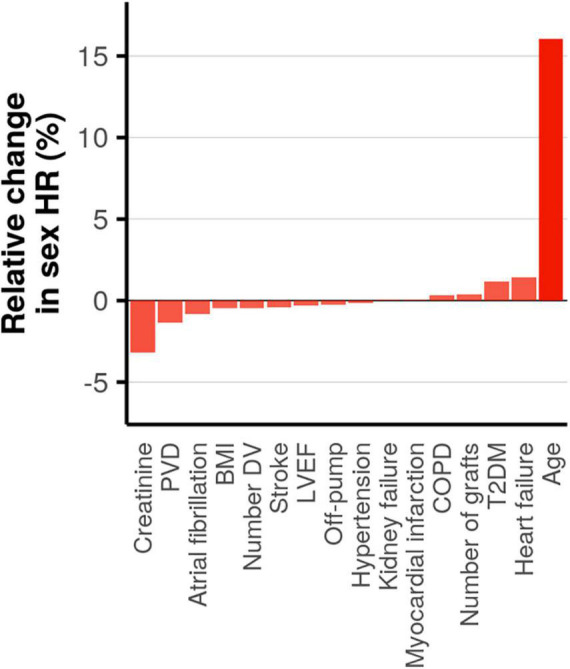
Assessing mediation by estimating the change in the hazard ratio (HR) for female sex on post-CABG survival when removing a covariate. Ordered from small to large, with opacity based on magnitude of change.

**FIGURE 5:**
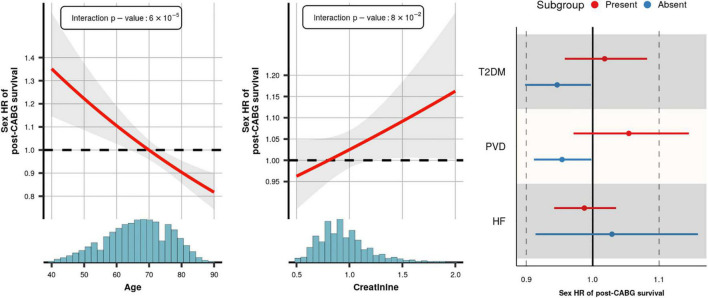
Subgroup specific effects of female sex on post-CABG survival. *x*-axis histogram provides the sample age distribution. Presented hazard ratio curve is based on the *imputed* model and accounts for the variables reported in the footnote of Figure 3. The interaction *p*-values for age and creatinine are provided in the illustration, for T2DM, PVD, and HF these were 0.07, 0.04, and 0.35, respectively. HR, hazard ratio; the interval and shaded area represent a 95% confidence interval.

As an initial step in quantifying the contribution of patient or procedure characteristics to the female HR we estimated pairwise correlations. The heatmap of the correlation coefficients (Supplementary Appendix Figure 4) showed limited interdependencies, indicating that most characteristics affect the female HR independently of one another. We subsequently determined the influence of each patient or procedure characteristic on the female HR using a leave-one-out analysis (Figure 4). The largest change of 16% was observed when removing age: female HR of 0.98 with age included vs. 1.13 with age removed. The next most influential patient characteristics were creatinine, PVD, heart failure, and type 2 diabetes; all less than 5% change. Next we estimated subgroup specific female HR effects for these five prioritized mediators, finding that post-CABG survival was longer (compared to men) in women without PVD (interaction *p*-value = 0.04), or women aged 70 or older (interaction *p*-value = 6 × 10^–5^). The age interaction additionally indicated that at younger women often had shorter post-CABG survival, although most of the events (in both men and women) occurred after 70 years; Figure 5 and Supplementary Appendix Figure 5.

We explored the validity of the proportional hazards assumption for female sex HR, finding no (*p*-value = 0.47) association between the Schoenfeld residuals and time. Furthermore, we performed a landmark analysis, removing participants with a follow-up less than 30 days (excluding 3,548 subjects of whom 1,985 died). The landmark analysis indicated that, after covariable adjustment, women were slightly protected against post-CABG compared to men HR 0.93 (95%CI 0.90; 0.97). Although, conform the non-proportional hazard test, confidence interval overlapped with the HR including all follow-up: 0.98 (95%CI 0.94; 1.02); Supplementary Appendix Figure 6.

## Discussion

In this individual patient data meta-analysis of five international cohorts we confirmed previous findings that women have a worse post-CABG survival rate compared to men: HR 1.20 (95%CI 1.16; 1.25). Accounting for differences in clinical characteristics at the time of CABG resulted in a neutral association (HR 0.98 95%CI 0.94;1.02); which was predominantly driven by difference at CABG presentation in age (age-mediation). Focussing on subjects with a 30+ day follow-up revealed a slight protective effect of female sex on post-CABG survival HR 0.93 (95%CI 0.90; 0.97) which did not significantly deviate from estimates using the entire follow-up period. Pre-planned subgroup analyses showed that, while on-average men and women did not differ much in their post-CABG survival after accounting for patient difference, older women (70+) or women without PVD survived longer than men. This was offset by a shorter female survival at a younger age.

This age dependency in long-term survival is especially relevant given the trend of women contributing more to the younger population of MI patients, observed in both the United States and Europe (14, 15). While short term (1 year) follow-up in younger women did not show changes in survival despite less guideline-recommended treatment, our data suggest that longer term survival is worse in these younger women compared to men (16). This survival difference between younger men and women seemed to be independent of other recorded clinical characteristics (indicated by a low Spearman’s correlation as provided in Supplementary Appendix Figure 5, and the leave-one-out analysis presented in Figure 4), such as type 2 diabetes, number of diseases vessels and whether the procedure was performed off-pump.

Our finding that younger women have higher post-CABG mortality than younger men may be explained by several factors. Biologically, sex differences in atherosclerotic disease are pronounced in young women where oestrogens may still play a role in the composition of the atherosclerotic plaque (17). In women, and specifically in those who have myocardial infarction at younger ages, plaque rupture is often not the cause of the coronary obstruction, but the event is triggered by “erosion” of the blood vessel wall (18). Eroded plaques may give rise to different symptoms as the blood clot often forms more gradually, which may result in late recognition of MI. Subsequent heart damage may be more severe, impacting long-term outcomes. Whether or not these erosions have been a substrate in the younger CABG women in these studies remains unknown. Additionally, the age dependent change in survival may be related to younger women present with a greater combination of risk factors than men, which decreases, or even reverses, with advancing age. This underscores the importance of studying young female MI patients, improving their care, and increasing the awareness of sex-specific symptoms. While sex differences in younger patients warrant considerable attention, we recognize that in about half our patient sample (median age 67) sex differences in long-term post-CABG survival were neutral or in favor of women (Figure 5 and Supplementary Appendix Figures 4, 5), and the majority of events occurred in older aged subjects.

Throughout the analyses there was considerable between country/study-heterogeneity, however random effects meta-analyses did not change results (Supplementary Appendix Table 4). Part of this observed between study heterogeneity might be due to actual study-specific difference in how men and women a treated after CABG, or potentially due to our pragmatic decision to focus on variables that were sufficiently frequently measured in participating studies, resulting in omitting some relevant predictors of post-CABG such as type of CABG procedure, or patient socioeconomics measures. Irrespective of the source of the observed between study-heterogeneity, accounting for this using random effects meta-analysis (Supplementary Appendix Table 4) did not impact results. Related, while we had access to individual participant data, a number of variables were unavailable or only partially available in specific studies. To explore this we employed a novel multi-level multiple imputation method (13), and carefully compared the sex HR of the *imputed* model to estimate from the *study-specific adjustment* model. The general agreement between the two approaches (HR 0.99 95%CI 0.95, 1.03 for the study-specific model vs. HR 0.98 95%0.94, 1.02 for the imputed model) suggest that the model choice and approach to missing data had little influence on results. While only 21% of our sample were women, due to the considerable overall number of 102,263 CABG patients, precision of our estimates was high. For example, while on average we did not find a significant difference between men and women in post-CABG survival after accounting for covariates, the 95% confidence interval suggest that should there be a difference, this is likely small and lies between a HR of 0.94 and 1.02. Finally, while we did not find evidence (using Schoenfeld residuals) of this in the current study, the sex association may be time dependent, and might change after even longer follow-up.

Current perspectives on mechanisms, diagnosis and treatment of cardiovascular disease are based on research and clinical evidence predominantly leveraged from male subjects. This is mainly due to the underrepresentation of women (<25%) in clinical trials for CAD (19). In this study, however, we have shown that sex differences in outcome after CABG surgery depend on the patient’s age and PVD status at procedure. The observation that women without PVD survive longer than men, is likely closely related to the stage of atherosclerosis, where at a later stage there is little difference between men and women (20).

## Conclusion

In conclusion, sex differences in post-CABG survival were readily explained by patient characteristics. Pre-planned subgroup analyses suggest that should there be any remaining difference between men and women, on-average women are likely to live longer than men after CABG, with the important caveat of considerable shorter survival in young women receiving CABG.

## Data Availability Statement

The data analyzed in this study is subject to the following licenses/restrictions: The individual participant data can be obtained through request to the study specific contributors, conditional on (inter)national regulations. Requests to access these datasets should be directed to individual contributers.

## Ethics Statement

Patients provided informed consent, and ethical approval was obtained through regional procedures from the institutional review boards in the respective centers. The Swedish Ethical Review Authority waived the need for informed consent, for the SWEDEHEART registry.

## Author Contributions

SH, FA, and HR contributed to the idea and design of the study. US, MH, DM, CSR, RB, HS, TK, LT, JS, CMR, FA, and HR contributed and curated their local databases. AFS performed the meta-analysis presented, had full access to all the data in the study, and takes responsibility for the integrity of the data and the accuracy of the data analysis, and drafted the manuscript. All authors critically revised the manuscript and approved submission.

## Conflict of Interest

MH has received honoraria from Idorsia not related to this study. The remaining authors declare that the research was conducted in the absence of any commercial or financial relationships that could be construed as a potential conflict of interest.

## Publisher’s Note

All claims expressed in this article are solely those of the authors and do not necessarily represent those of their affiliated organizations, or those of the publisher, the editors and the reviewers. Any product that may be evaluated in this article, or claim that may be made by its manufacturer, is not guaranteed or endorsed by the publisher.
