# Supplementary material for: Unravelling the Difference Between Men and Women in Post-CABG Survival
Source: Front Cardiovasc Med. 2022 Apr 13;9:768972. doi: 10.3389/fcvm.2022.768972 (PMC9043514; doi:10.3389/fcvm.2022.768972)
Supplement: Supplementary file 1 [file Data_Sheet_1.PDF]

LOTTERY: Appendix

1 Methods

All analyses were conducted in R[1], using the packages: survival[2], ggplot2[3], metafor[4], data.table[5], dplyr[6], wesanderson[7], pheatmap[8], and openxlsx[9].

2 Appendix Tables and Figures

Appendix Table 1: Percentage missings, stratified by study

Variable	Australia	Japan	Sweden	USA	Multinational
Sex	0.000	0.00	0	0.00	0.00
Off-pump	0.007	0.00	0	0.00	0.00
Age	0.020	0.00	0	0.00	0.00
BMI	0.306	0.14	0	0.26	0.69
Hypertension	0.002	0.00	0	0.00	0.00
T2DM	0.004	0.00	0	0.00	0.00
Creatinine	0.030	0.19	0	0.07	1.02
Myocardial infarction	0.004	0.00	0	0.00	0.00
Kidney failure	100.000	0.00	100	0.00	100.00
Number DV	0.030	0.00	100	0.06	0.00
Stroke	0.000	0.00	0	100.00	0.00
Heart failure	0.011	0.00	0	0.00	100.00
Atrial fibrillation	0.000	100.00	0	0.00	0.00
COPD	0.002	0.00	0	0.00	100.00
Number of grafts	0.000	100.00	0	100.00	0.00
PVD	0.004	0.00	0	0.00	0.00
LVEF	100.000	6.82	100	1.05	100.00
Death	0.000	0.00	0	0.00	0.00
Follow-up time	0.000	0.00	0	0.00	0.00

Note:

The Swedish study did provide LVEF as a factor

Appendix Table 2: Patient characteristics at the time of CABG procedure and outcomes; stratified by study and sex

	Australia		Japan		Multinational		Sweden		USA		
**Variable**	**Men**	**Women**	**Men**	**Women**	**Men**	**Women**	**Men**	**Women**	**Men**	**Women**	**Missing**
n	44837	11135	1590	537	2140	308	25100	6667	5585	1683	
Age (median [IQR])	66.00 [58.00, 73.00]	70.00 [62.00, 76.00]	68.00 [62.00, 75.00]	72.00 [65.00, 77.00]	60.00 [53.00, 68.00]	66.00 [58.00, 71.00]	67.00 [60.00, 74.00]	71.00 [63.00, 76.00]	65.29 [58.29, 72.02]	68.77 [61.37, 75.40]	0.0
BMI (median [IQR])	28.07 [25.35, 31.25]	28.31 [24.77, 32.72]	23.44 [21.50, 25.48]	22.97 [20.75, 25.33]	27.41 [25.06, 29.76]	27.34 [24.14, 31.16]	26.79 [24.66, 29.38]	26.99 [24.09, 30.12]	29.22 [26.06, 32.94]	29.76 [25.85, 34.62]	0.2
Creatinine (median [IQR])	0.90 [0.78, 1.04]	0.77 [0.64, 0.93]	0.92 [0.80, 1.19]	0.73 [0.60, 1.00]	0.98 [0.88, 1.11]	0.79 [0.69, 0.90]	1.00 [0.88, 1.16]	0.85 [0.72, 1.02]	1.00 [0.86, 1.20]	0.84 [0.70, 1.07]	0.1
Number of grafts (median [IQR])	5.00 [4.00, 7.00]	5.00 [4.00, 7.00]	NA [NA, NA]	NA [NA, NA]	3.00 [2.00, 4.00]	3.00 [2.00, 4.00]	3.00 [3.00, 4.00]	3.00 [2.00, 4.00]	NA [NA, NA]	NA [NA, NA]	9.4
Number DV (median [IQR])	3.00 [2.00, 3.00]	3.00 [2.00, 3.00]	3.00 [3.00, 3.00]	3.00 [3.00, 3.00]	3.00 [2.00, 3.00]	3.00 [2.00, 3.00]	NA [NA, NA]	NA [NA, NA]	3.00 [2.00, 3.00]	2.00 [2.00, 3.00]	31.9
LVEF (median [IQR])	NA [NA, NA]	NA [NA, NA]	60.00 [49.00, 67.60]	63.00 [52.00, 70.00]	NA [NA, NA]	NA [NA, NA]	NA [NA, NA]	NA [NA, NA]	55.00 [45.00, 60.00]	60.00 [50.00, 65.00]	90.8
Follow-up time (median [IQR])	4.61 [2.10, 7.74]	4.96 [2.29, 8.12]	5.21 [4.36, 5.94]	5.15 [4.39, 5.99]	2.73 [1.55, 3.55]	2.70 [1.42, 3.55]	6.84 [4.16, 9.14]	6.97 [4.49, 9.29]	3.00 [1.50, 4.75]	2.91 [1.42, 4.66]	0.0
Death (%)	3834 (8.6)	1232 (11.1)	315 (19.8)	86 (16.0)	32 (1.5)	8 (2.6)	4750 (18.9)	1448 (21.7)	477 (8.5)	175 (10.4)	0.0
Off-pump (%)											0.0
absent	3587 (8.0)	1054 (9.5)	599 (37.7)	197 (36.7)	1749 (81.7)	244 (79.2)	24227 (96.5)	6330 (94.9)	4380 (78.4)	1305 (77.5)	
present	41247 (92.0)	10080 (90.5)	991 (62.3)	340 (63.3)	391 (18.3)	64 (20.8)	873 (3.5)	337 (5.1)	1205 (21.6)	378 (22.5)	
missing	3 (0.0)	1 (0.0)	0 (0.0)	0 (0.0)	0 (0.0)	0 (0.0)	0 (0.0)	0 (0.0)	0 (0.0)	0 (0.0)	
Hypertension (%)											0.0
absent	9683 (21.6)	1701 (15.3)	283 (17.8)	71 (13.2)	1201 (56.1)	109 (35.4)	15700 (62.5)	3479 (52.2)	1022 (18.3)	228 (13.5)	
present	35153 (78.4)	9434 (84.7)	1307 (82.2)	466 (86.8)	939 (43.9)	199 (64.6)	9400 (37.5)	3188 (47.8)	4563 (81.7)	1455 (86.5)	
missing	1 (0.0)	0 (0.0)	0 (0.0)	0 (0.0)	0 (0.0)	0 (0.0)	0 (0.0)	0 (0.0)	0 (0.0)	0 (0.0)	
T2DM (%)											0.0
absent	29696 (66.2)	6487 (58.3)	812 (51.1)	243 (45.3)	1938 (90.6)	269 (87.3)	18888 (75.3)	4619 (69.3)	3522 (63.1)	881 (52.3)	
present	15139 (33.8)	4648 (41.7)	778 (48.9)	294 (54.7)	202 (9.4)	39 (12.7)	6212 (24.7)	2048 (30.7)	2063 (36.9)	802 (47.7)	
missing	2 (0.0)	0 (0.0)	0 (0.0)	0 (0.0)	0 (0.0)	0 (0.0)	0 (0.0)	0 (0.0)	0 (0.0)	0 (0.0)	
Myocardial infarction (%)											0.0
absent	21030 (46.9)	5231 (47.0)	1461 (91.9)	504 (93.9)	1293 (60.4)	198 (64.3)	11258 (44.9)	2839 (42.6)	3576 (64.0)	987 (58.6)	
present	23806 (53.1)	5903 (53.0)	129 (8.1)	33 (6.1)	847 (39.6)	110 (35.7)	13842 (55.1)	3828 (57.4)	2009 (36.0)	696 (41.4)	
missing	1 (0.0)	1 (0.0)	0 (0.0)	0 (0.0)	0 (0.0)	0 (0.0)	0 (0.0)	0 (0.0)	0 (0.0)	0 (0.0)	
Kidney failure (%)											90.6
absent	0 (0.0)	0 (0.0)	1484 (93.3)	512 (95.3)	0 (0.0)	0 (0.0)	0 (0.0)	0 (0.0)	5365 (96.1)	1604 (95.3)	
present	0 (0.0)	0 (0.0)	106 (6.7)	25 (4.7)	0 (0.0)	0 (0.0)	0 (0.0)	0 (0.0)	220 (3.9)	79 (4.7)	
missing	44837 (100.0)	11135 (100.0)	0 (0.0)	0 (0.0)	2140 (100.0)	308 (100.0)	25100 (100.0)	6667 (100.0)	0 (0.0)	0 (0.0)	
Stroke (%)											7.3
absent	40445 (90.2)	9840 (88.4)	1335 (84.0)	494 (92.0)	2096 (97.9)	301 (97.7)	22962 (91.5)	6023 (90.3)	0 (0.0)	0 (0.0)	
present	4392 (9.8)	1295 (11.6)	255 (16.0)	43 (8.0)	44 (2.1)	7 (2.3)	2138 (8.5)	644 (9.7)	0 (0.0)	0 (0.0)	
missing	0 (0.0)	0 (0.0)	0 (0.0)	0 (0.0)	0 (0.0)	0 (0.0)	0 (0.0)	0 (0.0)	5585 (100.0)	1683 (100.0)	
Heart Failure (%)											2.5
absent	39472 (88.0)	9368 (84.1)	1236 (77.7)	391 (72.8)	0 (0.0)	0 (0.0)	22288 (88.8)	5780 (86.7)	4738 (84.8)	1308 (77.7)	
present	5362 (12.0)	1764 (15.8)	354 (22.3)	146 (27.2)	0 (0.0)	0 (0.0)	2812 (11.2)	887 (13.3)	847 (15.2)	375 (22.3)	
missing	3 (0.0)	3 (0.0)	0 (0.0)	0 (0.0)	2140 (100.0)	308 (100.0)	0 (0.0)	0 (0.0)	0 (0.0)	0 (0.0)	
Atrial fibrillation (%)											2.1
absent	33445 (74.6)	8346 (75.0)	0 (0.0)	0 (0.0)	2034 (95.0)	289 (93.8)	23114 (92.1)	6190 (92.8)	5141 (92.1)	1553 (92.3)	
present	11392 (25.4)	2789 (25.0)	0 (0.0)	0 (0.0)	106 (5.0)	19 (6.2)	1986 (7.9)	477 (7.2)	444 (7.9)	130 (7.7)	
missing	0 (0.0)	0 (0.0)	1590 (100.0)	537 (100.0)	0 (0.0)	0 (0.0)	0 (0.0)	0 (0.0)	0 (0.0)	0 (0.0)	
COPD (%)											2.5

Appendix Table 2: Patient characteristics at the time of CABG procedure and outcomes; stratified by study and sex *(continued)*

Variable	Men	Women	Men	Women	Men	Women	Men	Women	Men	Women	Missing
absent	39573 (88.3)	9592 (86.1)	1555 (97.8)	519 (96.6)	0 (0.0)	0 (0.0)	23419 (93.3)	6000 (90.0)	4726 (84.6)	1340 (79.6)	
present	5264 (11.7)	1542 (13.8)	35 (2.2)	18 (3.4)	0 (0.0)	0 (0.0)	1681 (6.7)	667 (10.0)	859 (15.4)	343 (20.4)	
missing	0 (0.0)	1 (0.0)	0 (0.0)	0 (0.0)	2140 (100.0)	308 (100.0)	0 (0.0)	0 (0.0)	0 (0.0)	0 (0.0)	
PVD (%)											0.0
absent	39948 (89.1)	9833 (88.3)	1367 (86.0)	498 (92.7)	1996 (93.3)	280 (90.9)	22579 (90.0)	5835 (87.5)	4229 (75.7)	1177 (69.9)	
present	4889 (10.9)	1300 (11.7)	223 (14.0)	39 (7.3)	144 (6.7)	28 (9.1)	2521 (10.0)	832 (12.5)	1356 (24.3)	506 (30.1)	
missing	0 (0.0)	2 (0.0)	0 (0.0)	0 (0.0)	0 (0.0)	0 (0.0)	0 (0.0)	0 (0.0)	0 (0.0)	0 (0.0)	

Appendix Table 3: Study specific 10th percentile of the cummulative mortality time (years), with 95% confidence interval

strata	Years	Lower	Upper
Australia, Female	5.83	5.47	6.31
Australia, Male	6.71	6.53	6.93
Japan, Female	3.35	2.78	4.18
Japan, Male	2.78	2.43	3.17
Multinational, Female	NA	NA	NA
Multinational, Male	NA	NA	NA
Sweden, Female	4.76	4.52	4.99
Sweden, Male	4.82	4.68	4.96
USA, Female	3.75	3.13	4.19
USA, Male	4.17	3.75	4.51

Appendix Table 4: Random effect estimates of female sex on post-CABG survival

	HR (95%CI)
**Crude**	1.20 (1.06;1.35)
**Covariable adjusted**	1.01 (0.92;1.10)
**Covariable adjusted + imputation**	1.00 (0.91;1.10)

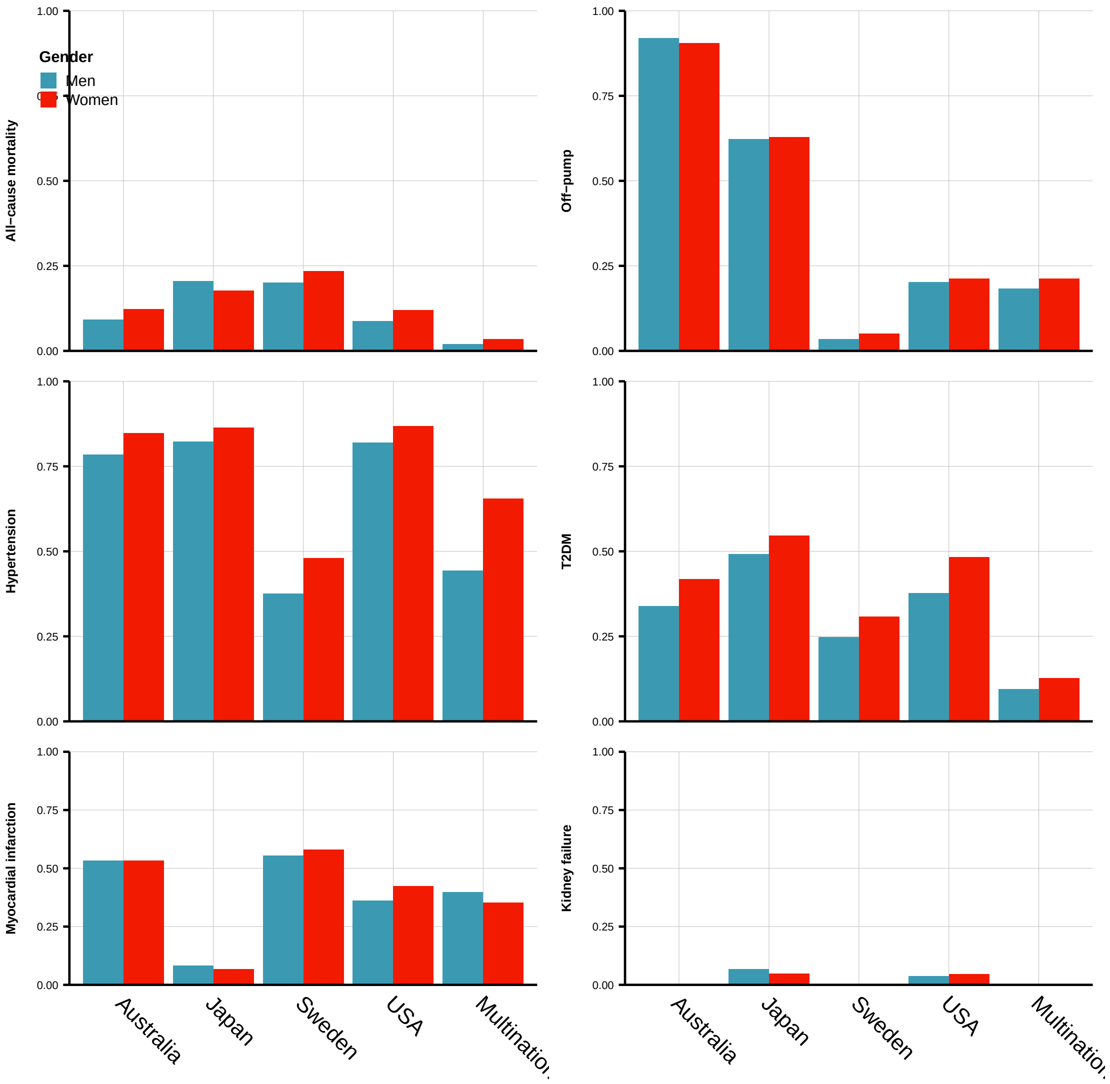

Appendix Figure 1: Patient Characteristics stratified by study and gender; empty columns indicate variables missing completely

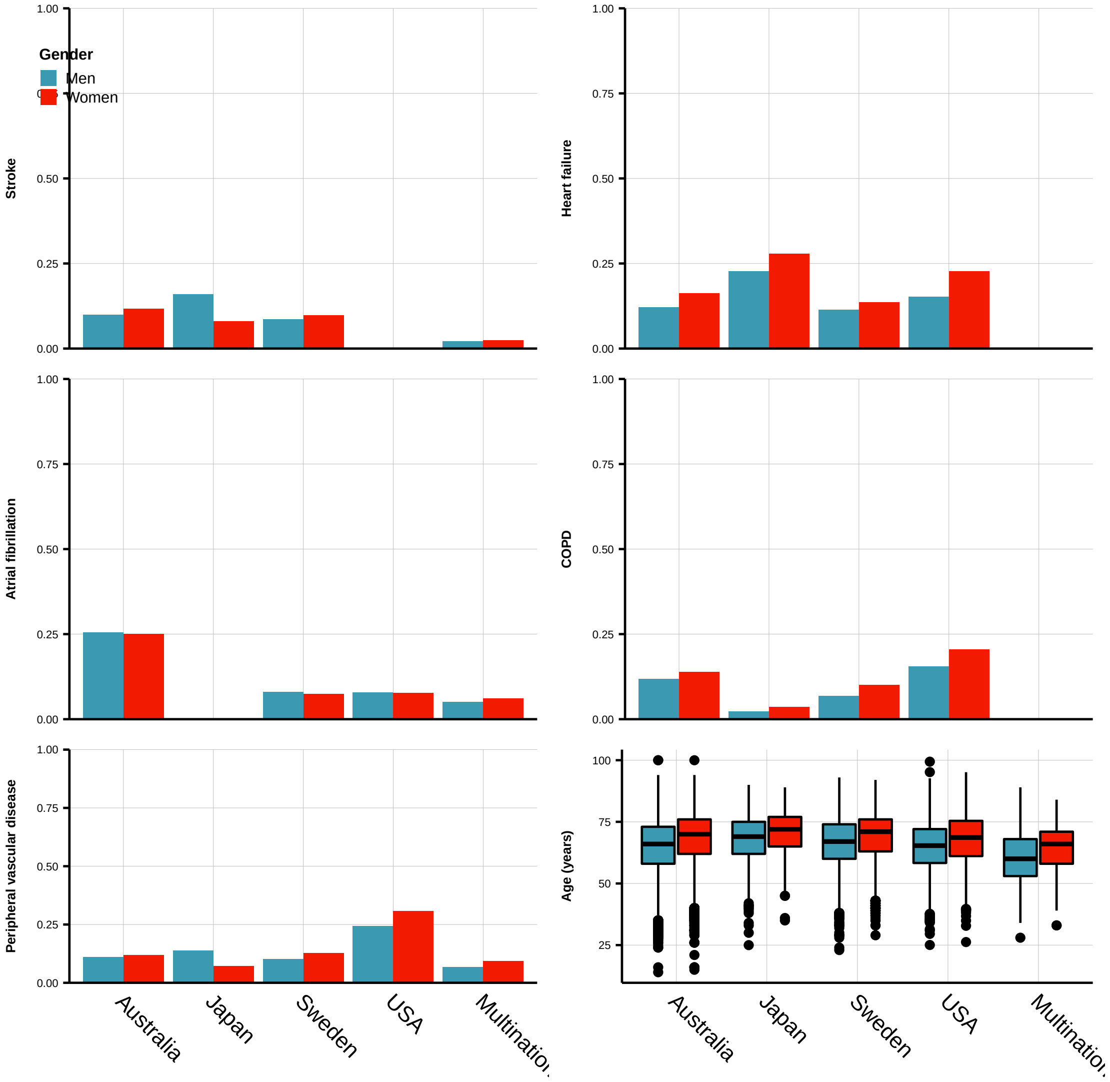

Appendix Figure 2: Patient Characteristics stratified by study and gender; empty columns indicate variables missing completely

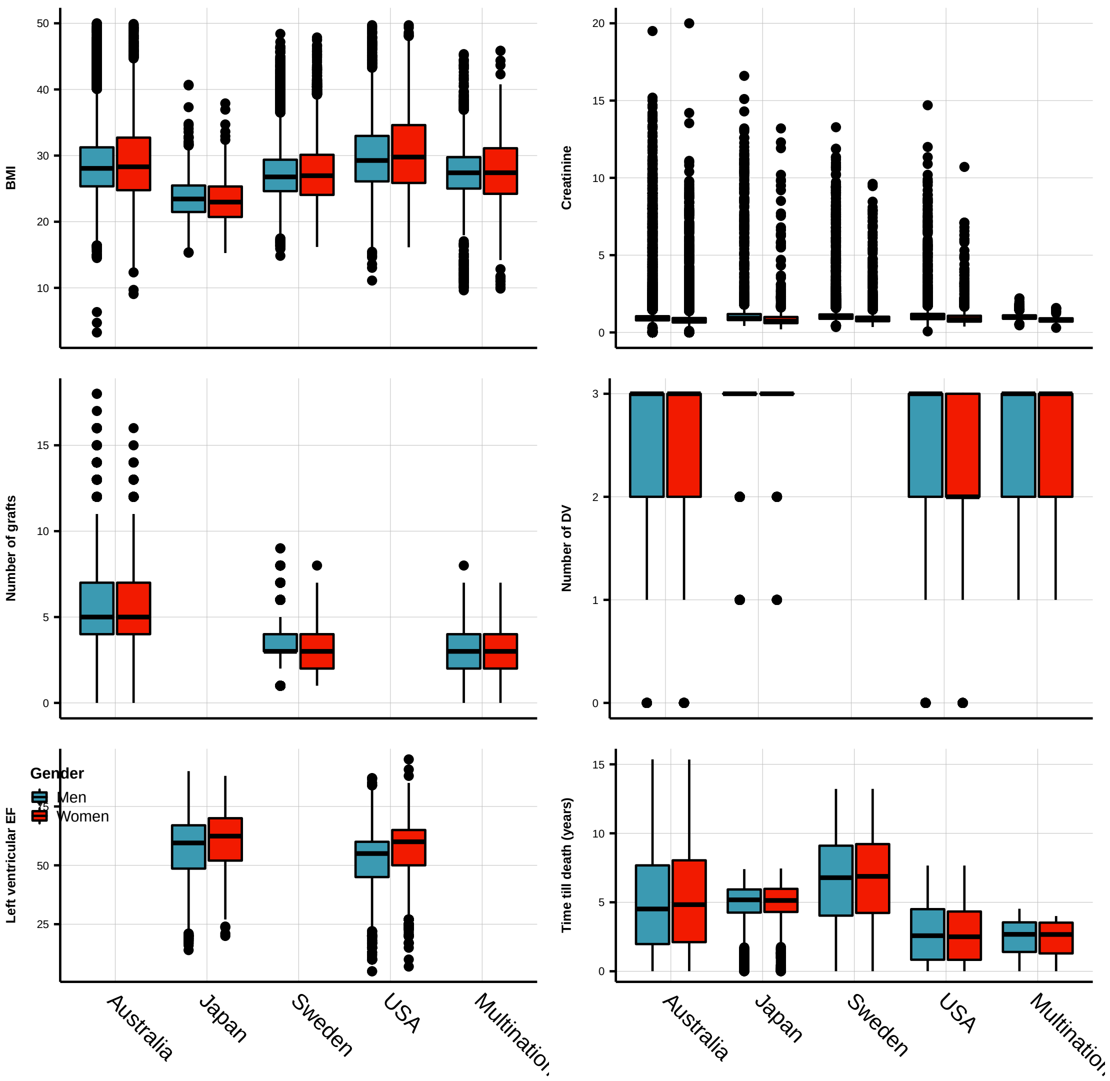

Appendix Figure 3: Patient Characteristics stratified by study and gender; empty columns indicate variables missing completely

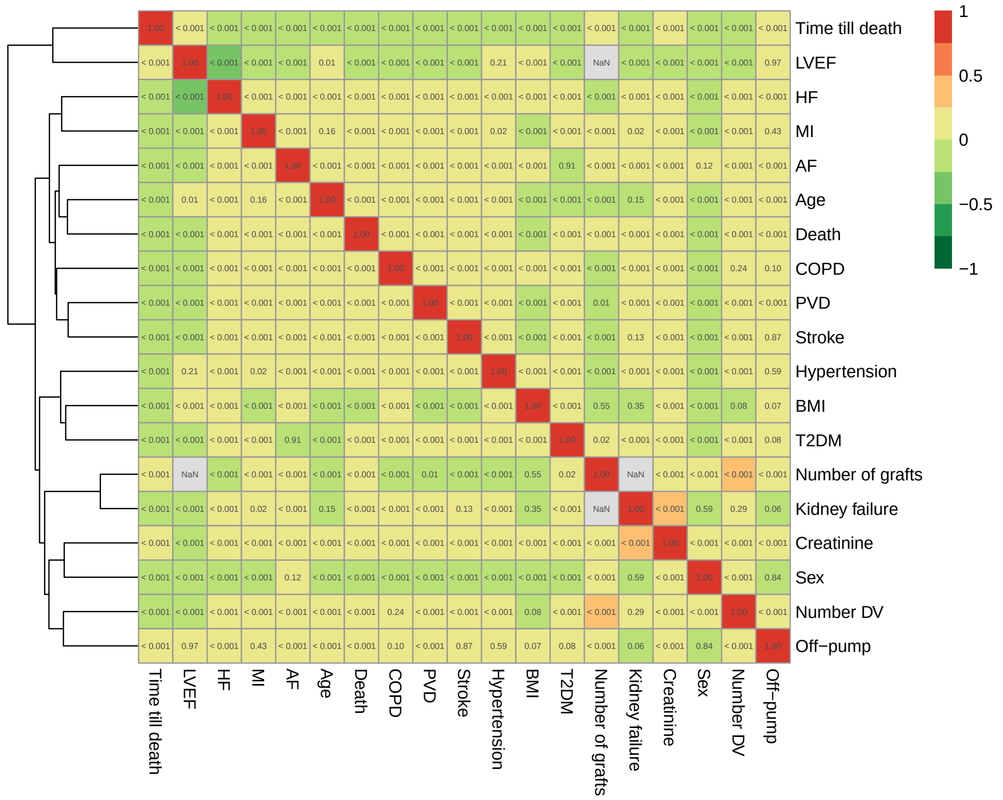

Appendix Figure 4: Spearman's pairwise correlation, with variables ordered by the y-axis dendogram. Numbers indicate p-values; Grey tiles indicates missing correlation coefficient; study specific correlation coefficient were meta-analyzed using Fisher's transformation.

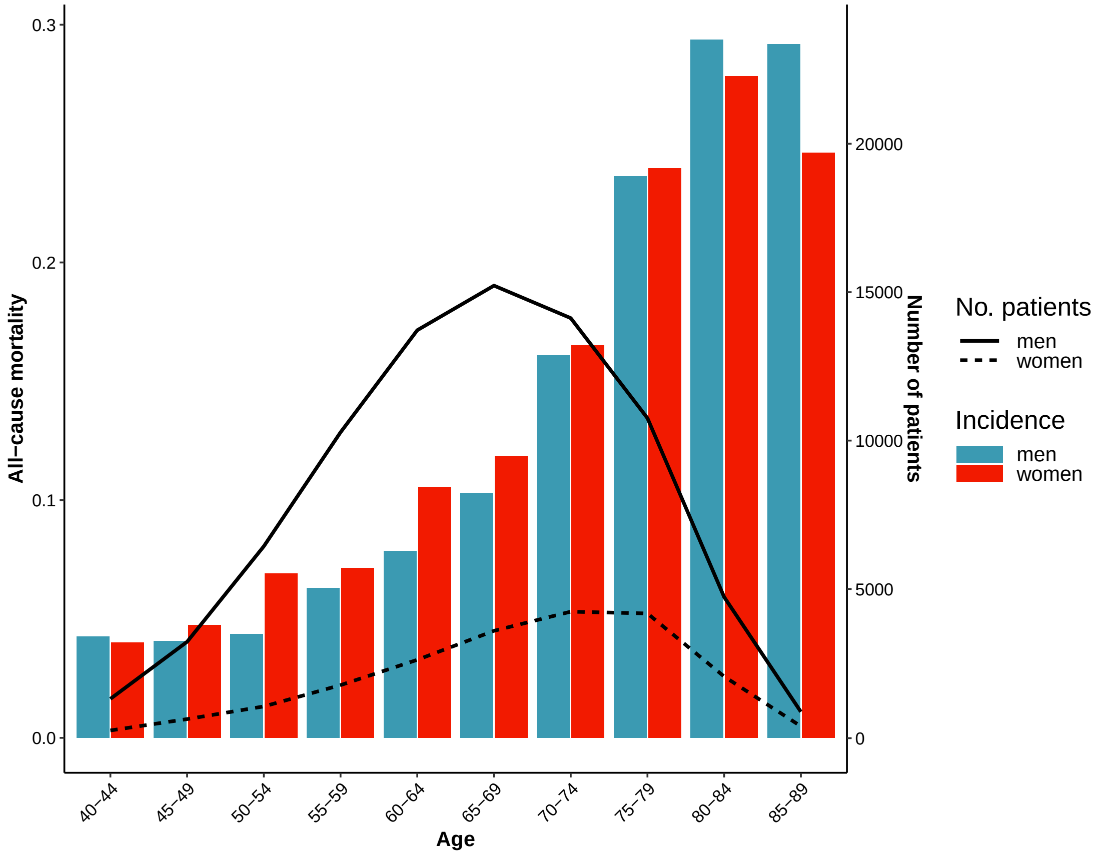

Appendix Figure 5: The post-CABG incidence of all-cause mortality stratified by age and sex, with the right y-axis and lines indicating total number of patients. Estimates were not adjusted for patient characteristics or procedure differences.

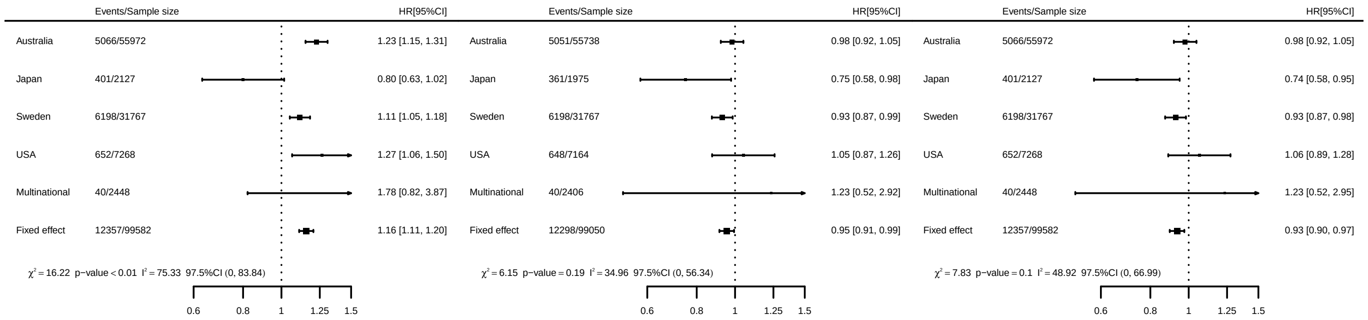

Appendix Figure 6: A landmark analysis of the female sex HR of post-CABG survival focussing on particiants with at least 30 days of follow-up.

References

[1] R Core Team. *R: A Language and Environment for Statistical Computing*. R Foundation for Statistical Computing. Vienna, Austria, 2018. URL: https://www.R-project.org/.

[2] Terry M Therneau. *A Package for Survival Analysis in S*. version 2.38. 2015. URL: https://CRAN.R-project.org/package=survival.

[3] Hadley Wickham. *ggplot2: Elegant Graphics for Data Analysis*. Springer-Verlag New York, 2016. ISBN: 978-3-319-24277-4. URL: http://ggplot2.org.

[4] Wolfgang Viechtbauer. “Conducting meta-analyses in R with the metafor package”. In: *Journal of Statistical Software* 36.3 (2010), pp. 1–48. URL: http://www.jstatsoft.org/v36/i03/.

[5] Matt Dowle and Arun Srinivasan. *data.table: Extension of ‘data.frame‘*. R package version 1.11.8. 2018. URL: https://CRAN.R-project.org/package=data.table.

[6] Hadley Wickham, Romain François, Lionel Henry, and Kirill Müller. *dplyr: A Grammar of Data Manipulation*. R package version 0.7.6. 2018. URL: https://CRAN.R-project.org/package=dplyr.

[7] Karthik Ram and Hadley Wickham. *wesanderson: A Wes Anderson Palette Generator*. R package version 0.3.6. 2018. URL: https://CRAN.R-project.org/package=wesanderson.

[8] Raivo Kolde. *pheatmap: Pretty Heatmaps*. R package version 1.0.12. 2019. URL: https://CRAN.R-project.org/package=pheatmap.

[9] Alexander Walker. *openxlsx: Read, Write and Edit XLSX Files*. R package version 4.1.0. 2018. URL: https://CRAN.R-project.org/package=openxlsx.
